# Recent advances in dairy goat products

**DOI:** 10.5713/ajas.19.0487

**Published:** 2019-07-18

**Authors:** Lucia Sepe, Anastasio Argüello

**Affiliations:** 1 CREA Research Centre for Animal Production and Aquaculture, Bella Muro 85051, Italy; 2 Animal Production and Biotechnology Group, Institute of Animal Health and Food Safety, Universidad de Las Palmas de Gran Canaria, Arucas, Las Palmas 35413, Spain

**Keywords:** Goat, Dairy Products, Technology, Packaging, Health Properties

## Abstract

Goat population world-wide is increasing, and the dairy goat sector is developing accordingly. Although the new technology applied to the goat industry is being introduced slowly because the weight of traditional subsector in the dairy sector, considerable advances have been made in the last decade. Present review focuses on the emerging topics in the dairy goat sector. Research and development of traditional and new dairy goat products are reviewed, including the new research in the use of goat milk in infant formula. The research in alternatives to brine, production of skimmed goat cheeses and the use of different modified atmosphere packaging are also addressed. Special attention is given to antibiotic residues and their determination in goat milk. Functional foods for human benefits are a trending topic. Health properties recently discovered in dairy goat products are included in the paper, with special attention to the antioxidant activity. The dual-purpose use of goats by humankind is affecting the way of how new technology is being incorporated in the dairy goat sector and will certainly affect the future development of dairy goat products.

## OVERVIEW OF THE WORLD DAIRY GOAT PRODUCTION

The earliest evidences for cheese making date about 7,500 years ago [1]. Archaeological sieve fragments were found in Kuyavia, Poland, showing lipids residues from cow milk. It is acknowledged that goat was one of the earliest domesticated animals for milk production and human benefits (8,000 B.C.) [2]. It is argued that the first cheeses were made from goat and sheep milk. The importance of goat milk changed as cow milk was introduced into human diets, and consequently its economic significance/impact decreased progressively in the world. Goat milk differs from cow milk in its higher digestibility, diverse alkalinity, higher buffering capacity and confident therapeutic use in human nutrition. Goat milk has smaller fat globules; distinct protein’s polymorphism, with significantly lower as_1_- and higher as_2_-casein, thus higher tolerability than cow milk; when acidified, shows better digestibility than cow milk. Goat milk has more short- and medium-chain fatty acids, giving a special ability to provide energy mostly for growing children. To date goat milk accounts for less than 1.6% of the dairy production worldwide [3].

Most of goat milk is consumed fresh, especially in those regions where goat is reared for family consumption, e.g., in Asia, Africa and South America [4]. Thus, the official statistics related to goat milk and dairy production are scarce and likely underestimated, as is their importance for human nutrition.

In 2017 dairy goats were estimated at 217.7 million heads (i.e., 21% of the total goat population), mostly distributed in Asia (52%) and Africa (39%), with the remainder in Europe, Americas, and Oceania [3]. Goat milk world production accounted 18.66 million tons, where India is the top producer in the world, with more than 6.16 million tons of goat milk, followed by Bangladesh, Sudan, Pakistan, France, Greece, Turkey, and Spain respectively.

Breed, breeding program (i.e. seasonal or year-round), production system, feeding system, organization of the production chain, research and development activity are main factors affecting goat milk production and quality.

**Figure 1:**
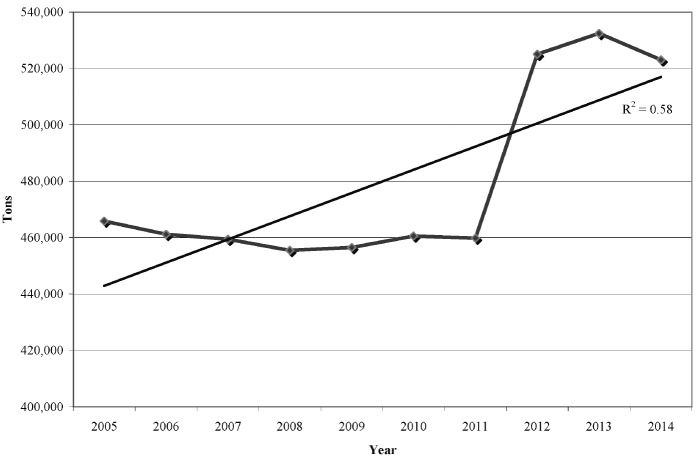
Trend of the world goat cheese production in the decade 2005 through 2014 [3]. After a nearly steady production until 2010, the world goat cheeses production showed a rapid increase up to 2013. Latest official data available year 2014 [3].

Goat dairy products around the world consist of yogurt, fermented milk, curd and cheese, ice cream, made of pure goat milk or mixed with other types of milk (i.e., sheep, cow). According to latest FAOSTAT database [3], in 2014 the world production of goat cheeses counted totally 523,000 tons, showing a rapid increase from 2011 to 2013 (Figure 1), just over 15%, in comparison to world cow dairy production in the same period (5.4%).

**Figure 2:**
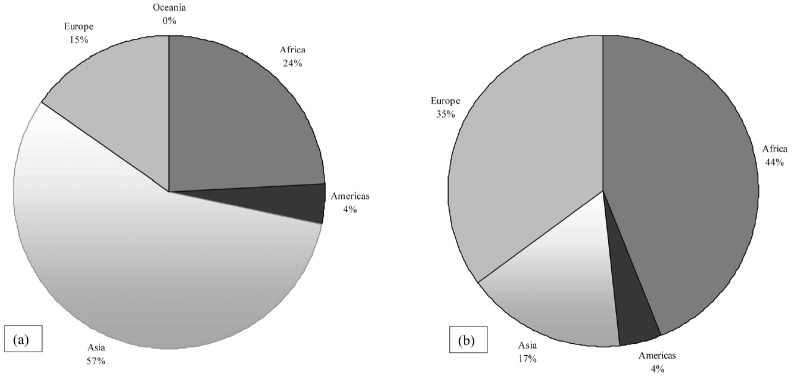
Fresh goat milk production (a) (latest official data available year 2017) and cheese production (b) (latest official data available year 2014) (%) per world region [3]. The variation in milk production between 2014 and 2017 was 1 to 2 points percentage. Thus the comparison between fresh milk production 2017 and cheese production 2014 is considered.

Despite a large proportion of goat milk in the world was produced in Asia (10.55 million tons, i.e. 56% in 2014, and 57% in 2017), the highest production of goat cheese came from Africa, in particular from South Sudan (110,750 tons). Europe produced 35% of world goat cheese with only 15% of goat heads. This may be explained with the differences in production system, extensive vs. more intensive, and the destination of milk, drinking milk rather than cheese (Figure 2).

The world areas top producers of goat milk cheese were Eastern Africa (i.e. South Sudan), and Western Europe (87,407 tons, from France, Germany, and Austria), followed by Northern Africa (85,105 tons, from Sudan, Morocco, and Tunisia) and Southern Europe (81,854 tons, from Greece, Spain, Italy, Portugal, Albania, and Malta).

Goat milk production systems vary considerably by region. In Asia, Africa and South America, the majority of goat rearing is carried out by small enterprises, farmstead and/or family operations, which have not access to modern pasteurization facilities. Raw milk is processed or sold locally to dairies that process daily, thus eliminating the risks of bacterial contaminations and spoilage during storage and transportation. In fact, these operation scales are so small that often official statistics of goat milk and dairy products are not kept in many countries. The production system is extensive, mostly with grazing and rarely with feed supplementation. In other regions, such as North America, goat milk production is increasing slowly but steadily. According to the U.S. Department of Agriculture, as of the beginning of 2017 there were 373,000 registered dairy goats in the United States, with the largest numbers in Wisconsin (44,000 heads) and California (41,000 heads). Similarly, dairy goat operations in the U.S. are mainly of small and farmstead scales. Industrial scale goat farms and milk processing plants have been established in recently years due to an increased interest and demand among consumers.

The present review aims to present the recent advances in dairy goat production, with a multidisciplinary approach.

## CURRENT STATUS OF GOAT DAIRY PRODUCTS

### Traditional dairy products

Accordingly to Gellynck and Kühne [5], traditional dairy products can be defined those, with or without label, that meet four criteria: i) the key production steps of a traditional cheese product must be performed in a certain area, which can be national, regional or local; ii) the traditional cheese must be authentic in its recipe (ingredients), origin of raw material, and/or production process; iii) the traditional cheese must be commercially available for at least 50 years; and iv) it must be part of the gastronomic heritage.

Goat cheese is a universe itself, where each type of cheese has its own “character”, a restricted trait among the cheeses. Traditional goat cheeses are characterized by diversity of appearance, typical sensory profile, different process and tools, and ripening degree, such as fresh vs ripened lactic cheese, fresh or ripened curd, moulded, spreadable, cooked or uncooked, natural caves, etc.; some of them are linked to specific breeds of goats, such as the Italian Fatulì cheese, produced only with milk from Bionda dell’Adamello goat breed (PAT - Traditional Agri-Foodstuffs, Italian Quality Label).

Generally, goats are the only domesticated animals that can feed with restricted vegetation in harsh environments where steep gradients, damp climate, thin limestone based or rocky soil represent difficult condition for agricultural activities by human.

Few studies gather specifically the traditional goat dairy products worldwide. The “Atlas of goat products” [6] is the only worldwide collection giving information on origin, type, area and technique of production, scale of production, uses and demand trend on dairy goat products. This inventory reports 170 goat dairy products, among which 150 cheeses, and drinking milk, yogurt, ice cream, salted whey from 23 countries in 4 continents. It is surely not an exhaustive list (Table 1). An update is encouraged to include the products not yet inventoried, the estimation of production and the latest recognition labels applied.

Broadly the traditional dairy goat products are linked to the “environment”. Similar to wine production, they are the result of the “*terroir*”: mankind as for *savoir faire*, cultural heritage, and environment, such as soil, water availability, location, climate, vegetable and animal biodiversity.

The dairy products by natural starters in milk are commonly located in very dry and warm climate areas such as desert regions. Goat breeding in these areas has been the main occupation of numerous people since ancient times, and goat’s milk and cheese have always been a fundamental part of their economy, diet and cultural heritage. The products may be consumed fresh or dry. Examples are J’ben from Morocco, Djamid/Jameed, and Labaneh from the Middle East. The small scale of production supplies the market locally but diffusely (Table 1). In Turkey [7], the traditional goat dairy products are destined to local and regional market, such as Erzincan, Izmir Brined, and Cimi Tulum cheeses (tulum means wrapped in goatskin), Karin Kaymagi, Ezine, Carra or Testi, Sepet, Kefir, Hellim cheese (the equivalent of Halloumi from Cyprus), and Ayran cheese.

The cheeses from enzymatic coagulation, with animal or vegetal rennet, represent the most of the world production, both farmstead and industrial. The farmstead scale is linked to the low amount of production and kind of process, both labelled and non-labelled cheeses. Few traditional cheeses are produced at an industrial scale, with one exception of Greek Feta protected designation of origin (PDO) cheese.

El Galiou et al [8] carried out a chemical and microbiological survey on 28 fresh raw goat’s milk cheeses from different small domestic producers in Northern Morocco. This study compared three manufacturing processes: calf rennet, with and without cooling milk after milking, and vegetable rennet without cooling milk after milking. The variability in pH values (3.8 to 4.0) and dry matter contents (24.9 to 31.2 g/100 g), together with the free fatty acids and microbiota composition, revealed a large variability of milk quality and lack of standardization in the manufacturing process.

Martinez et al [9] conducted a review in which the description of 32 Spanish goat cheeses, their manufacture processes, their organoleptic characteristics and the studies carried out on them were reported (Table 2). Raynal-Ljutovac et al [10] presented an up-date on the French market for goat milk cheeses. It is suggested that the particular technology of lactic cheeses and the slow coagulation at room temperature are perfectly suited with habits of women herding little flock of goats in late 19th century. These processes became so famous abroad that they are synonymous with French type cheese.

In Europe, the traditional products are protected and valorised by the recognition with labelling system, where the production is regulated by severe disciplinary. The labels are namely the PDO and the protected geographical indication (PGI): PDO cheese is produced and processed and prepared in its area of production; PGI is produced or processed or prepared in its area of production, and both benefit from a long-lasting good reputation. As of April 2019 European Union had 216 PDO cheeses and 59 PGI cheeses [11]. Among them, 41 were from goat milk (Table 3).

It was found [12] that the PDO labelling created a quantifiable added-value, in comparison to other cheeses, roughly +50% when sold at the market and +20% on farm. The PDO label system is best fitted with the small scale production of traditional goat cheeses and helps the distribution of the value along the whole production chain, and to increase the power of producers’ supply in the market.

Due to the increased popularity, many cheeses originally made with pure goat milk are made with cow milk or a mix of goat, sheep and cow, such as Añejo Enchilado cheese produced in Mexico, Caprino cheese in Italy, and Halloumi cheese in Cyprus. Sometimes the rennet originally used (from kid and lamb) has been replaced by liquid calf rennet, and consequently mild flavour cheeses are produced, which are preferred by today’s consumers. For example, the Norwegian brown whey cheese Brunost is less consumed, while the popularity of rennet- and acid-coagulated cheeses has increased [13]. The Turkish Gokceada cheese in the past was made with a home-made kid rennet, however, commercial calf rennet is commonly used today [14].

The dairy production in South America is the result of the adaptation of imported technologies to local customs and environment. The widespread small and farmstead scale productions of Queso blanco, Queso fresco, Requeson, and Quesillo in Venezuela, Peru and Bolivia, respectively, indicate that traditional products are critically important to local economies; moreover they are characterized by the women labour. On the other hand, Queijo Minas Frescal produced in Brazil is an exception to the role, as it is produced by specialized industries.

The traditional cheeses are appreciated for their original shape, sensory profile, and the occasion of maintaining gastronomic and rural tradition. Darfyieh and Arichi cheeses from Lebanon may be considered among the most original cheeses: they are produced from milk and whey, respectively, and then put in alternate layers into a goat skin to ripen. The production is ancient, linked to the Baladi goat breed. The Sudanese white *Gibna Bayda* cheese is produced by women, like many other cheeses, and is representative of many traditional cheeses in Africa: no standard processing methods, resulting in high variability in nutritional composition, microbial proliferation of natural lactic acid bacteria (LAB), and sensory characteristics [15].

The world dairy production claims several traditional milk derived products: yogurt (naturally probiotic) and not -enzymatic coagulation curds. Tamime et al [16] reviewed the panorama of goat fermented milk, where most products are traditionally made locally in different countries in the Middle East and in other regions, and some are produced at industrial scale. The fermented milk and yogurt change name by country and region, and the quality is affected by the local strains of LAB (traditional and adjunct, including probiotic organisms), yeasts and moulds (or combination of these) employed. Among the products, they described Caprine yoghurt, the Italian Gioddu, the Chinese Tarag, caprine probiotic yoghurt, kefir, the concentrated yogurt Labneh, the salted yoghurt Tuzlu and Shankleesh, dried fermented milks such as Kurut, Keş, Kiş.

Pandya and Ghodke [17] reviewed several studies on cream, butter such as Turkish “Yayik” butter, more appreciated than sheep or cow milk butter, chhana and paneer, very popular in India and nearby countries. Chhana is a heat and acid coagulated Indian milk product, generally processed by women. It is obtained from adding sour whey from previous day to hot milk (70°C). The coagulum is filtered through fine cloth, pressed and used immediately. Paneer is a variation of chhana, a soft uncured cheese, more similar to Cottage or Queso blanco. Paneer is traditionally produced with cow milk, little from goat milk.

### New products

Cheese and yogurt are predominant products of goat milk, however, there are other economically viable products derived from goat milk as well. For this review we will focus on recent advances in ice cream, fermented beverages and infant formula.

The addition of fortification substrates to ice cream made from goat milk was addressed by Chaves et al [18]. The substrates evaluated were chia mucilage and bean gum, which both impacted moisture and viscosity. Adding polydextrose (as a prebiotics) and *Lactobacillus paracasei* subsp. *paracasei* and *Bifidobacterium longum* + *Bifidobacterium bifidum* in a combined culture (as probiotic cultures) to goat ice cream yielded very interesting results extending the shelf life of the product [19]. Relatedly, viability of *Lactobacillus acidophilus* and *Bifidobacterium* BB-12, [20] in goat ice cream was increased (increasing the value as a probiotic) by addition of Carob extract and whey powder, with best results when the substances were included at 1%.

Fermented goat milk products containing *Lactobacillus acidophilus* LA-5, *Bifidobacterium animalis* subsp. *lactis* BB-12 and *Propionibacterium jensenii* 702 were made by Ranadheera et al [21], with no negative effects on the sensory properties. Collagen hydrolysate, cheese whey, and acai pulp (*Euterpe oleracea*) have been successfully incorporated in fermented goat milk beverages to extend the viability of probiotics [22]. SHIME is a unique scientifically validated dynamic model of the complete gastrointestinal tract for study of physicochemical, enzymatic, and microbial parameters in the gastrointestinal tract in a controlled in vitro setting. Using SHIME, [23] was able to keep *Lactobacillus rhamnosus* and *Streptococcus thermophilus* viable during their passage through the intestinal tract, which had a positive effect on gut microbiota metabolism using a grape probiotic fermented beverage made with goat milk. Use of inulin and oligofructose as prebiotics in goat milk fermented beverages including *Bifidobacterium lactis* has had favourable effects enhancing probiotic viability and sensory characteristics [24].

After the approval of the use of goat milk or derivates in infant formulas (Directive 2013/46/EU), research in this area has been conducted. With simulation of infant digestive conditions, similar results were observed for cow and goat milk, although production of bioactive peptides differed [25]. In reference to the presence of oligosaccharides in goat milk versus cow colostrum, [26] have showed that oligosaccharides from goat milk were determined as more potential ingredients of infant formulae based on potency of adherence inhibition, safety, and the availability of the starting material. Furthermore, [27] compared in vitro proteolysis of human, cow, and goat milk, and observed more similarities between human and goat milk than human and cow milk.

## RESEARCH ADVANCES IN DAIRY GOAT PRODUCTS

### Technology process and packaging

The adoption of new technology in the dairy goat sector is usually slower than that in the dairy cattle sector, largely because of the lesser world economic importance and relatively lower input production systems of the dairy goat industry world-wide.

Presently diets low in salt and fat are viewed as having positive effects on human nutrition and health. This is in part relevant to the making of some types of goat cheese because of brine usage. Relatedly, Miloradovic et al [28] attempted to reduce the NaCl level of a white brined cheese from 6% to 3% but noted a marked decrease in consumer acceptability, apparently because of an altered texture profile and an increased cohesiveness. Further studies in this area are needed to solve such consumer acceptability issues. Regarding the level of fat, goat cheese made with skimmed milk has posed some negative characteristics for consumers [29]. New technologies, such as supercritical fluid extraction, hold promise to decrease the level of fat after cheese is produced [30].

Considerable scientific attention is being given to microbiome and microbiota, including the dairy goat sector. Using cheese as a probiotic carrier has been proposed by Meira et al [31]. These authors incorporated *Bifidobacterium animalis subsp. lactis Bb-12* and *Lactobacillus acidophilus* La-05 into goat Ricotta with little change in physical-chemical characteristics, mainly derived from a lower concentration of lactose due to a higher transformation to lactic acid. It was reported that Ricotta serviced as a carrier of the probiotic, providing protection for survival of the stressful conditions imposed by the human gastrointestinal tract.

Biotechnology is now being applied in practical settings, an example of which is widespread use of substances extracted from bacteria, yeast and fungus. However, use of such products in the dairy goat sector is not common. *Listeria monocytogenes* is a problem in raw milk cheeses around the world, and its control is key factor. The use of bacteriocin-like inhibitory substances have been proposed to control the growth of *Listeria monocytogenes* in goat cheese [32]. This research group used *Enterococcus durans* E204 as a source of bacteriocin-like inhibitory substances that successfully inhibit the *Listeria monocytogenes* growth in Jben goat cheese (a traditional Moroccan fresh cheese).

Storing cheese frozen as a means of conservation has long been a common practice in the cheese industry and new knowledge has emerged in the last decade [33,34]. Although the length of time of cheeses in freezers has always been a concern, Park [34] did not observe any major negative effects of freezing goat milk Monterey Jack cheese at −20°C for 5 years on consumer acceptance. Conversely, Andic et al [33] reported negative effects of freezing Motal cheese on organoleptic quality (i.e., free fatty acids released).

There has been some research attention to packaging of dairy foods in the last 10 years. Technological characteristics of foils for packaging of cheese during ripening have been recently determined [35]. Foils differing in permeability to CO_2_ and O_2_ have influence on appearance and physical characteristics of cheese that consequently affect the final quality of products. Relatedly, use of different atmospheres during the packaging was addressed by [28]. To replace the O_2_ in the atmosphere surrounding the cheese has an impact on the ripening process. It was determined that replacing the normal atmosphere with a modified one, e.g., 60% CO_2_ and 40% N_2_, reduced the growth of psychrotrophic bacteria and yeasts/moulds, although no differences were observed in proteolysis or cheese colour. Arvanitoyannis et al [36] found different results with other gas levels of 40% CO_2_, 55% N_2_, and 5% O_2_; 60% CO_2_ and 40% N_2_; and 50% CO_2_ and 50% N_2_. Vacuum packaging has been widely used in the last 40 years; it has been reported that this type of packaging has little or no effects after ripening of Urda whey cheese [37]. Future research is needed to identify optimal combinations of gas levels for specific types of cheese and the use of biodegradable plastic materials.

### Residual antibiotics

Antimicrobial resistance is a growing global health concern in both human and animals. An increasing number of studies has shown that antimicrobial usage in humans [38] and animals [39] is the main driver of the development of antimicrobial resistance, although an environmental role has been recently proposed as well [40]. Antibiotic residues in animal foods are caused by antimicrobial usages in animal treatments and feed additives. Obviously, this area is of importance to the dairy goat sector and has consequently received considerable research attention.

Screening tests are commonly used in detecting antibiotic residues in milk. For detecting oxytetracycline residues in goat milk, Charm Rosa and SNAP tests have performed satisfactorily [41]. Betastar Combo, SNAP Betalactam, SNAP Tetracycline, and Twinsensor tests have been successfully used in goat milk [42]. Brilliant black reduction test AiM, Delvotest MCS, Eclipse 100, and Copan Milk tests have been also used in goat milk for detection of residues of non-beta-lactam and beta-lactam antibiotics, with concerns expressed by the authors about their sensitivity for non-beta-lactam and no concerns for beta-lactam [43,44]. Also the successful use of electronic nose (a metal oxide semiconductor gas-sensing device) have been proposed for Penicillin G residues detection [45].

The presence of antibiotic residues in cheese has been evaluated recently [46]. Goat milk was added with European Union maximum residue limits of amoxicillin, benzylpenicillin, cloxacillin, erythromycin, ciprofloxacin, enrofloxacin, and oxytetracycline to manufacture ripened Spanish Tronchon cheese. It was reported that high concentrations of enrofloxacin and ciprofloxacin residues were observed in the cheeses after 60 d of maturation. Therefore, special attention must be implemented to guarantee the safety of cheese in regard to antibiotic residues. A different approach using whey as an indicator was used by Giraldo et al [47]. Results were similar to those of Quintanilla et al [46], where different antibiotics varying in retention behaviour in curd.

The off-label use of some drugs in the goat sector is common because of the small number of products labelled for use with goats. Off-label use of macrolides in dairy goats was addressed by Quintanilla et al [48]. There were no residues of erythromycin, tylosin or spiramycin after the mandatory 7 days of withdrawal. In regard to use of antibiotics in reproductive management practices, no residual antibiotics were found in milk when doxycycline, oxytetracycline, and sulfathiazole-framycetin were used at recommended doses in intravaginal sponges [49].

The presence of antibiotic residues in milk did not markedly impact the manufacturing process of dairy goat products, including yogurt [50] and cheese [46], but different results could be observed in relation to the antibiotic type.

Microbial inhibitor tests are widely used in laboratories for screening antimicrobials in milk because they are easy to use and inexpensive [51]. However, some substances present in milk can interfere with the test. No interference of antiparasitic drugs such as albendazole has been observed [51]. On the contrary, ivermectin residues in goat milk after parasite treatment altered the microbial inhibitor test [52]. The doses of the antiparasitic drug play an important role, with [53] observing that high doses interfere with the most microbial inhibitor tests including BRT MRL, Delvotest SP-NT MSC, and Eclipse 100. Residues of detergents used in the cleaning process of the milking machine can have impact on microbial inhibitor test results, depending on the chemical nature of detergent [54]. The presence of colostrum in milk samples interferes with normal use of microbial inhibitor tests due to the relatively high protein concentration [55]. Therefore, these authors conclude that microbial inhibitor tests are not suitable to detect antibiotic residues in colostrum.

### Health properties of dairy goat products

Bioactive compounds in food associated with proteins, lipids and polysaccharides are one of the most recent research fields, together with studies on their metabolism to assess their contributions to human health [56]. Scientific evidences stimulated a new consumers approach towards foods: the awareness that food may be more than basic nutrition but a step towards well-being. Research evidence and consumer awareness have elevated the functional food market in the entire world.

The main trait of goat milk that has contributed to its increasing interest by the consumers is its lower allergenic properties due to the lower level of α-_s1_-CN and its higher digestibility, linked to the higher proportion of short and medium chain fatty acids than cow milk [57]. In regions dominated by the consumption of cow milk, goat milk is known to be an alternative, especially in case of poor digestibility or intolerance to cow milk. However, one of the main limits by the consumer, besides the cultural heritage, is goat milk’s sensory profile.

While health properties [58,59] and utilization of particular bioactive compounds for childhood nutrition [60] of goat milk are increasingly studied, studies on goat milk dairy products are relatively scarce.

Goats grazing in pastoral systems, by eating large amounts of natural browsing plants, are almost unknown carriers of health promoting compounds. Goat dairy products supply in different way and measure the milk nutritional compounds and other new molecules: fatty acids, organic acids, exopolysaccharides (EPS) [61], vitamins, bioactive peptides and lipids, and enzymes.

Hereby, the recent advances on healthy contribution of goat dairy products are reviewed.

#### Lipid fractions in dairy products

World goat rearing is characterized mostly by grazing system, where animals feed pasture resources with low or no supplementation. Several studies found how the feeding system and the botanical composition of the pasture affect the nutritional profile of cheese in terms of fatty acid profile and content. Recently, Sant’Ana et al [62] evaluated the fatty acid profiles and sensory profiles of goat milk and Coalho cheese. Their study compared goats grazing Caatinga plants on native semiarid pasture (PS) (Brazil) or being raised in Confinement System (CS). Both cheese and milk from PS had slightly higher polyunsaturated fatty acids and saturated fatty acids ratio (PUFA/SFA) and a markedly lower atherogenic index and n−6/n−3 ratio, which are favourably considered for the potential impact on consumer’s health. The decrease in short-chain fatty acids (SCFA) in PS, in comparison to CS, was mostly due to the decrease of medium chain FA C12:0 and C14:0, both considered hypercholesterolaemic FA, resulting in the decreased atherogenic index (C12:0, from 3.26 to 2.59, and C14:0 from 10.86 to 9.05 in CS and PS, respectively). Furthermore, they found a reduction of short-chain saturated FA C6:0, C8:0 and C10:0, considered negative for the healthy profile of cheese. Trans-FA C18:2c9t11 (i.e., conjugated linoleic acid or CLA) was significantly lower in PS cheese (0.33% vs 0.38% total FA), whereas C18:3n−3 was significantly higher (0.23% vs 0.10% total FA), contributing to favourably decrease the ratio n−6/n−3.

In a semi-extensive system, goat flocks are fed forage and concentrate supplementation, mostly in certain lactation periods and physiological stages. Claps et al [63] reported significant differences in cheeses from Mediterranean red goats fed 8 Mediterranean forage species, 4 legumes and 4 grasses given in pureness, on fatty acid profile. Cheeses made with milk from goats fed *T. incarnatum*, *Triticosecale* and *H. vulgare* showed the highest content in CLA, while cheeses from goats fed *Vicia sativa* displayed the lowest CLA content but the highest content of alpha-linolenic acid (ALA), probably due to the higher level of tannic polyphenols in forage, which was able to interact with lipolysis and PUFA bio-hydrogenation. The highest health-promoting index was observed in cheeses from goats fed *H. vulgare* and *A. sativa*.

The lipid components of cheese, such as CLA, PUFA and polar lipids, may exert anti-inflammatory activities [64]. Traditional Greek Ladotiri and Kefalotiri cheeses were studied for their lipid fractions, in particular those able to inhibit platelet activating factors (PAF, a potent thrombotic phospholipid mediator of atherosclerosis). Cheeses showed inhibitory properties against PAF-induced platelet aggregation, and sphingomyelin, phosphatidylcholine and phosphatidylethanolamine lipid derivatives as the most biologically active compounds. The anti-inflammatory activities increase during lipolysis (during yogurt and cheese manufacture processes). Megalemou et al [65] found the most vigorous PAF inhibitors in yogurt from sheep and goat milk, in comparison to those from cow milk. The studies showed that native pastures as well as farm-grown forages can be considered effective low-cost feed to improve goat milk fatty acid composition without affecting yield, a pathway toward the sustainable intensification of goat production.

In an attempt to improve health properties of dairy goat products, trials were carried out to study the effect of vegetable oils and fatty acid supplementation on goat cheese nutritional profile. De Medeiros et al [66] experimented diets containing 4% vegetable oils (faveleira, sesame, or castor oils), taking also into account the sensory profile. The diets containing faveleira and sesame oils positively affected the fatty acid profile, increasing the unsaturated fatty acid (USFA) content, 35.73% and 35.08%, respectively, to levels considered beneficial to human health.

The studies on functional properties of bioactive compounds are applied mostly *in vitro* and in animal model, while still few studies concern human trial. Santurino et al [56] enriched naturally goats cheese by feeding Murciano-Granadina goats with extruded linseed with good consistency of retention of the bioactive ingredients in order to be applied in human clinical trials among overweight and obese subjects engaged in cardiovascular risk prevention study. Diet, besides increasing milk yield, significantly favoured the increase of CLA (2.32% vs 1.47% of total fatty acid methyl esters and 5-fold higher omega-3 (4.05% vs 0.49%), thus reducing 7-fold the ratio omega-6/omega-3, with beneficial effect for human health. ALA increased (0.64 g/100 g vs 0.13 g/100 g cheese), allowing the claim for “source of omega-3”, according to EU Regulation.

#### Angiotensin-converting-enzyme inhibitor peptides

Few studies reported on the presence of angiotensin-converting-enzyme (ACE) inhibitor peptides in goat cheese and yogurt. These enzymes, produced by hydrolysis of milk proteins by LAB, showed antihypertensive activity. In commercial Kefir made with goat milk, two peptides, with sequences PYVRYL and LVYPFTGPIPN, resistant to a simulated digestion were found with potent ACE inhibitor activities [67]. In one Spanish pure goat cheese and in Cabrales cheese [68], the peptide DKIHP (fragment β-CN f[47–51]) showed the highest ACE inhibitor activity, with the lowest half maximal inhibitory concentration (IC_50_) value as 113.1 μM.

#### Antioxidant capacity

Few studies were concerned the antioxidant molecules and capacity in goat dairy products. Several compounds exert antioxidant activity, protecting lipids and other molecules against the oxidation or the production of free radicals, which have been regarded as dangerous forerunner of cardiovascular diseases (CVD).

Sepe et al [69] fed Siriana goats with fresh *Avena sativa* in pureness to study the occurrence of phenolic compounds in milk, cheese and whey. They found a variety of phenolic compounds carried from milk to Caciotta cheese: 15 simple phenols, 5 benzoic acid derivatives and 5 unclassified phenolic compounds. Notwithstanding the presence in milk, cinnamic acid derivatives, hippuric acid, riboflavin, and indole derivatives were not found in cheese. One of the indole derivatives found in milk was recovered in cheese. These preliminary results allowed proposing a method to analyse phenolic compounds in cheese and to observe that a variety of phenolic compounds are carried from milk to whey and cheese, boosting the knowledge on the healthy benefits from goat dairy products.

In a more recent study [70], feeding system (free-range vs confinement), heat treatment (raw vs pasteurized) and season (dry vs rainy) were the variables on the antioxidant capacity and total phenolic content (TPC) in goat cheeses. Cheeses from goats grazing five hours in thorny deciduous forest land, dominated by shrubs, and supplemented (concentrate, hay, and silage), showed significantly higher antioxidant capacity compared with cheeses from confined feeding system. The same occurred for TPC, and in particular during dry season and from unpasteurized milk (363.21 mg gallic acid equivalent/L milk).

#### Lactic acid bacteria and probiotic dairy goat products

Recently, the interest for non-bovine healthy dairy products involved the research and dairy industry, conjugating the benefit traits of goat milk to the health properties of probiotic LAB. Besides the protective and technological properties, LAB produce or increase specific bioactive compounds in food.

Feta, probably the most famous traditional Greek cheese, made with blend of sheep and goat milk (up to 70:30 ratio), was produced with *Lb. plantarum* T571 strain, with the aim to investigate the probiotic performance as a co-starter [71]. Surviving *Lb. plantarum* was counted in probiotic Feta in levels required for claiming a probiotic effect (≥6 log colony-forming unit/g) during the storage at 4°C and ripened for 9 months, compared to the usual period by the industry (6 months). Moreover, the probiotic Feta was evaluated with similar or better scores of sensory attributes (i.e. acidity, sweetness, rancidity and hardness). Even though the consumer approach is predictable to complete the holistic evaluation, the authors considered the *Lb. plantarum* strain T571 as a promising candidate to develop functional Feta cheese.

Several studies [72] demonstrated that goat milk is a successful carrier for probiotic LAB, in cheese, yogurt, fermented milk, milk beverage, ice-cream and other products, since these dairy products can facilitate probiotic viability and high surviving level during storage. Dairy goat products exhibit a high potential in heightening the functional properties of probiotics such as gastrointestinal tolerance and adhesion to intestinal epithelium. On the other hand, certain LAB strains cause concerns in the sensory profile of dairy products, as they are capable of producing volatile aromatic compounds that contribute to an elevated sensory profile, sometimes too “goaty” for the consumer.

A recent analysis [73] highlighted the critical role of autochthonous LAB from in the protective, technological and functional properties of dairy goat products. In particular, EPS from LAB such as *Lb. plantarum*, under regular consumption, are associated with antimutagenic property, while antigastritis, antiulcer, and antitumor effects are associated with EPS from another LAB. Moreover, autochthonous strains of *L. coryniformis* are good producers of cobalamin (vitamin B_12_) while *Lactococcus lactis* is a good source of riboflavin (vitamin B_2_) and folate.

## THE FUTURE

### Perspectives of dairy goat products

The large variability, in physical-chemical and microbiological quality, in those environments where the hygienic conditions of milking and cheese-making may lead to high bacterial load, is ascribable to lack of standardization in the manufacturing process. Moreover, the need for improving milk quality and/or using heat-treated milk to produce the cheese is common, besides the implementation of strategies to enhance the chemical and microbiological quality of traditional products [8].

The dairy goat industry has gathered the desire of the consumer, in slow but steady growth, for products alternative to cow’s milk. Innovative goat dairy products are therefore increasing, with ingredients that can satisfy the taste of today’s consumer [24], such as inulin in beverage, or with technologies that respect the original ingredient in traditional cheeses [74].

The dairy industry has also beheld the chance to produce functional dairy products from goat milk, which may potentially benefit human health and contribute to attenuate issues related to CVD, obesity and diabetes. One of the most emergent challenges for the dairy industry is to counteract the image of milk by-products as cholesterol enemy. In this way, one of the perspectives is linked to the cholesterol-lowering properties exerted by some LAB [75]. Seven strains of LAB, used in goat yogurt and cheese process with raw milk, were found to be able to decrease the cholesterol first in broth and then in cheeses during ripening. It was reported that the cholesterol-lowering property was more significant in cheeses ripened for 60 days than 30 days, with the exception of *Lb. plantarum* VS166 and VS513. Further studies on autochthonous LAB and their strains may lead to novel functional dairy goat products or improved traditional products.

One of the greatest challenges for humankind is to combat the climate change, which prompts us to develop sustainable strategies to adapt to the changes in water availability, temperature, soil system, vegetable and animal biodiversity, both preserving the environment and satisfying the increasing food demand. Goat is, in many ways, the most adaptable species that allows sustainable breeding and production, especially in those harsh environments characterized by scarce resources. Moreover, goats emit less methane than other domestic ruminants, contributing to the mitigation of climate changes [76]. In this sense, there is a reasonable perspective that goat rearing and dairy production will continue to grow and develop in the future, provided that sustainable strategies mentioned above are developed.

Furthermore, given the growth of market demand for goat products, the system must be equipped with advanced, fast and yet accurate methods to detect adulteration of fraudulent blends of goat’s milk from other milks. Advances in electronic nose have made it a rapid, accurate and not invasive tool to detect adulterations with high sensitivity and specificity. Several studies have validated its accuracy and cost-effectiveness of this method, using a large database library [77]. This approach has also shown encouraging potential in other applications, such as discrimination of watered milks, differentiation of diet supplementation, identification of cheese ripening age/stage, in the near future.

Recent issues that characterize the demand by the postmodern consumer concern “all-natural product”, less processed and preservative-free options, all favouring the growing of the demand for organic products. The world trend for organic foods is increasing: the global organic retail sales in 2017 counted 92.08 Billion € (about 103.81 Billion $), having grown about 2.7-fold in the last decade [78]. The global organic cheese market is expected to increase 14% by 2023 [79], with Europe and China to be key future markets, whereas India and Brazil to be emerging contributors to this growth. The organic food, that implies prohibition for artificial additives and genetically modified organisms, is perceived as a healthier alternative to products from intensive system. Given the pastoral system that characterizes the goat breeding and production in most regions worldwide, organic goat production may be economically important in those regions where intensive system is dominant. Perspective is a small but steady growth.

### Strengths and features for enhancing milk production and consumption

The seasonality, which characterizes goat milk production, in certain states may be considered a limit, i.e. for industrial scale, while in artisan and farmstead scale it is considered a *plus* value, a factor that gives uniqueness to regional and traditional products. In fact, season means diversity of natural feedstuff (for botanical and chemical composition), climatic conditions and milk composition.

Breed in some regions is the key factor for sustainability and quality production. The native breeds, in fact, have the resilience, more than other breeds, to adapt themselves to the climatic change and to use the local resources more efficiently. One of the strengths of the native breeds lays in this nature. Only a few goat dairy products are linked to a native breed, as seen previously, but these cases could represent winning examples of biodiversity enhancement, worthy of being extended to other products. Furthermore, the label system attesting the origin (PDO and PGI) [12,73,80] has shown to be also a mean for supporting and safeguarding the maintenance of such rural identity, through the perpetuation of cheese making techniques. This label scheme, moreover, mostly for small and farmstead scale, clearly favours the quality instead the quantity growth, the preservation of local employment in rural far-flung areas and highlights a product in consumer’s eyes.

Many factors affect the quality of dairy products, i.e. feeding, breed, process (rennet, tools, technology, ripening), autochthonous LAB (that have the further property to differentiate the sensory profile), production system (environment and persons). Hence, one strategy to promote the increase of the demand for traditional products would be the multidisciplinary approach, in order to communicate their benefits and cultural heritage. One of the approaches may be the measurement of life cycle assessment, but still few studies concern goat dairy production system.

Finally, one of the most recent challenges is giving an economic measure to the ecosystem services performed by shepherds and breeders cheese-makers. This incentive system would represent a concrete recognition towards those intangible services they carry out in favour of safeguarding the biodiversity, maintaining the environment and cultural heritage.

## CONCLUSION

This article, after an overview on the worldwide traditional and new goat dairy production, presented the recent advances under different points of view: technology, packaging, residual antibiotics, and health benefits.

Dairy goats, once reared as the little cows for poor people or in harsh environments, are still one of the most efficient animals in bio-valorising the feed resources, mostly in difficult regions where goats cannot be replaced by other species, able to give quality meat, milk and hair/skin. It is affirmed particularly today, when the intensification of cow milk production signs the crisis of milk in many regions, and human population shows increasing level of intolerance and allergy towards cow milk. Additionally, when climatic changes sound the alarm asking humankind for new and more respectful approaches to the Nature, dairy goats represent a more viable and sustainable production system.

The research is giving answers to the popular belief about the health properties of goat’s milk products, common in many regions of the world. Nevertheless, further efforts are needed to highlight the results, together with further clinical studies on their potential effects on consumer health. In the future starting today, supplementary research in sustainably feeding goats, improving milk and by-products’ quality, eco-friendly and smartly exploiting milk whey, will be strategic for the development of the dairy goat production chain.

## Figures and Tables

**Table 1:** Main traditional goat cheeses and dairy products by regions of Africa, Middle East, Americas and Asia

Region	Country	Name	Milk (breed)/other dairy prod.	Scale of production	Ref.
Africa	Algeria	Djben/Jben/Gibneh	Raw goat, sheep, cow	Small	[7]
	Egypt	Dhani	Raw goat	Small	[7]
		Karish	Raw goat, camel	Small/Bedouins	[7]
		Mish	Raw goat, cow, camel	Small, home	[7]
	Morocco	J’ben	Raw goat	Small	[7]
	Sudan	Gibna Bayda	Raw goat	Farmstead	[18]

Middle East		Djamid/Jameed	Raw goat, sheep	Small	[7]
		Labaneh	Goat, cow, sheep/conc. yogurt	Small/Bedouins	[7]
	Lebanon	Darfyieh	Raw goat/cheese in goat skin	Small	[7]
		Arichi	Whey cheese/in goat skin	Small	[7]
	Turkey	Tulum (Erzincan, Izmir Brined and Cimi)	Raw goat	Nom.& transhumance s.	[8]
		Ayran	Raw goat	Nom.& transhumance s.	[8]
		Carra or Testi	Raw goat	Nom.& transhumance s.	[8]
		Ezine	Raw goat	Nom.& transhumance s.	[8]
		Hellim	Raw goat	Nom.& transhumance s.	[8]
		Karin Kaymagi	Raw goat	Nom.& transhumance s.	[8]
		Kefir	Raw goat	Small, Industrial	[8]
		Kurut, Keş, Kiş	Goat/dried fermented milk	Small	[19]
		Sepet	Goat	Small	[8]
		Tuzlu and Shankleesh	Goat/dried salted yogurts	Small	[19]
		Yayik	Goat/butter	Small	[21]

Americas	Argentina	Quesillo	Raw goat (Criolla)	Small	[7]
	Bolivia	Quesillo	Raw goat	Small	[7]
	Brazil	Minas frescal	Raw goat	Industrial (’70)	[7]
		Doce de leite	Goat/processed dessert	Small	[7]
	Mexico	Fresco [Queso]	Raw goat	Small	[7]
		Fresco de Aro	Raw goat	Small	[7]
		Ranchero de cabra de Queretaro	Raw goat	Small	[7]
		Ranchero molido	Raw goat	Small	[7]
	Peru	Quesillo	Raw goat	Small	[7]
		Queso Fresco	Raw goat	Small	[7]
	Venezuela	Queso Blanco	Raw goat (Baladi)	Small	[7]
		Natilla	Goat and cow/condensed	Small	[7]

Asia	India	Dahi	Raw goat/yogurt	Small	[20]
	China	Tarag	Goat/yogurt	Small	[19]

Nom. & transhumance s., Nomadic & transhumance systems.

**Table 2:** Spanish goat milk cheeses, by region and origin of the milk (adapted from [11])

Cheese name	Region	Milk	Goat’s breed
Acehuche	Extremadura	Goat	
Albarracín	Aragón	Goat	
Alhama de Granada	Andalusia	Raw goat	
Alicante	Comunitat Valenciana	Pasteurised goat	
Aracena	Andalusia	Raw goat	
Armada	Castile-León	Raw goat	
Babia-Laciana	Castile-León	Raw goat	Local breeds
Badaia	Basque Country	Raw goat	
Buelles	Asturias	Pasteurised goat	
Cádiz and Málaga	Andalusia	Pasteurised goat	Malagueña
Camerano PDO	La Rioja	Pasteurised goat	Serrana or Montesa
Cassoleta	Comunitat Valenciana	Goat/sheep/also cow	
Cendrat del Montsec	Catalonia	Raw goat	Murciana- Granadina
Conejero	Canary Islands	Goat	Majorera
Garrotxa	Catalonia	Goat	Alpina, Saanen, Malagueña, M. Granadina
Gata-Hurdes	Extremadura	Goat	Retinta
Gomero	Canary Islands	Goat/mixed with sheep or cow	Canaria
Gredos	Castile-León	Goat	
Herreño	Canary Islands	Goat/mixed with sheep or cow	Canary breeds
Ibores PDO	Extremadura	Goat	Verata, Serrana, Retinta
La Nucia	Comunitat Valenciana	Goat/sheep/also cow	
La Siberia	Exremadura	Raw goat	
La Vera	Extremadura	Goat	Verata
Los Beyos PGI	Asturias/Castile León	Goat/sheep/cow	
Majorero PDO	Canary Islands	Goat	Majorera
Murcia/Murcia al Vino PDO	Murcia	Pasteurised goat	Murciana,¬ Granadina
Palmero PDO	Canary Islands	Raw goat	Palmera
Quesaílla	Extremadura	Goat	
Servilleta	Comunitat Valenciana	Goat/sheep/cow	
Sierra Morena	Andalusia	Goat	Florida
Tenerife	Canary Islands	Goat	Majorera, Tinerfeña
Valdeteja	Castile-León	Raw goat	Local breeds

PDO, protected designation of origin; PGI, protected geographical indication.

**Table 3:** Traditional and new European goat cheeses (100% goat or blends) by EU label ) (adapted from [6,11,80])

Country	EU label1)	Cheese name						
Cyprus	PDO	Halloumi/Hellim (*Ap.*)						

France	PDO	Banon	Chabichou Du Poitou	Charolais	Chevrotin	Crottin De Chavignol	Mâconnais	Pélardon
		Picodon	Pouligny-Saint-Pierre	Rocamadour	Sainte-Maure De Touraine	Selles-Sur-Cher	Valençay	Brousse Du Rove (*Ap.*)
	PGI	Tomme Des Pyrénées						
	None	Boxe Cheese From Poitou	Cabecou D’autan	Cabecou Perigord	Calenzana (whey cheese)	La Feuille Du Limousine	Mothais Sur Feille	

Germany	PDO	Altenburger Ziegenkäse						

Greece	PDO	Anevato	Batzos	Feta	Formaella Arachovas Parnassou	Galotyri	Kalathaki Limnou	Kasseri
		Katiki Domokou	Kefalograviera	Ladotyri Mytilinis	Metsovone	San Michali	Sfela	Xygalo Siteias/Xigalo Siteias
		Xynomyzithra Kritis	Arseniko Naxou (*Ap.*)					
	PGI	Krasotiri Ko - Tiri Tis Possias (*Ap.*)						
	None	Anthotiros	Armirotiri	Chlorotiri	Galomyzithra	Kathoura	Kefalotiri	Kopanisti
		Manoura	Manouromizithra	Mizithra	Telemes	Xynotiri		

Italy	PDO	Formaggella del Luinese	Robiola di Roccaverano	Valle D’Aosta Fromadzo				
	PGI	Canestrato Di Moliterno						
	None	Agrino Orobie	Blue	Cachat	Cacioricotta	Canestrato D’aspromonte	Caprino della Carnia	Caprino della Val Vigezzo
		Caprino Semicotto	Caprino Valsesiano	Casieddu	Caso Conzato	Caso Peruto	Cevrin Di Coazze	Crotonese
		Fatulì	Felciata	Formaggio morbido della Valle D’Aosta	Jama	Juncata	Musulupu	Nicastrese
		Robiola del Bec	Tomino di Talucco	Vecjo di Cjavre	Ricotta (whey cheese)	Scuete (whey c.)		

Norway	None	Ekte geitost (whey c.)	Gudbrandsdalsost	Kvit geitost (whey c.)	Snoefrisk			

Poland	None	Blekitna kraina	Czarnuszka	Ser podpuszczkowy wedzony	Ser twarogowy swiezy	Ser zolty	Blekitna kraina	Ser twardy wedzony

Portugal	PDO	Queijo de Cabra Transmontano/Transmontano Velho	Queijo Rabaçal	Queijo Amarelo da Beira Baixa/Queijo Picante da Beira Baixa				
	PGI	Queijo mestiço de Tolosa						
	None	Algarve						

Sweden	None	Vit geitost	Mesost (whey c.)					

Switzerland	None	Ziegenkäse						

1) PDO, protected designation of origin; PGI, protected geographical indication; Ap., applied; whey c., whey cheese.
